# Cognitive dysfunction in animal models of human lewy-body dementia

**DOI:** 10.3389/fnagi.2024.1369733

**Published:** 2024-07-22

**Authors:** Caroline Haikal, Graham M. Winston, Michael G. Kaplitt

**Affiliations:** ^1^Weill Cornell Medicine, Department of Neurological Surgery, New York, NY, United States; ^2^Aligning Science Across Parkinson’s Collaborative Research Network, Chevy Chase, MD, United States

**Keywords:** lewy body dementia, Parkinson’s disease, Parkinson’s disease dementia, dementia with lewy bodies, preformed fibril, animal models

## Abstract

Cognitive impairments are a common feature of synucleinopathies such as Parkinson’s Disease Dementia and Dementia with Lewy Bodies. These pathologies are characterized by accumulation of Lewy bodies and Lewy neurites as well as neuronal cell death. Alpha-synuclein is the main proteinaceous component of Lewy bodies and Lewy neurites. To model these pathologies *in vivo*, toxins that selectively target certain neuronal populations or different means of inducing alpha-synuclein aggregation can be used. Alpha-synuclein accumulation can be induced by genetic manipulation, viral vector overexpression or the use of preformed fibrils of alpha-synuclein. In this review, we summarize the cognitive impairments associated with different models of synucleinopathies and relevance to observations in human diseases.

## Introduction

Parkinson’s Disease Dementia (PDD) and Dementia with Lewy Bodies (DLB) are two of the most common pathologic causes of dementia. DLB is the second most common pathologic cause of dementia, affecting up to 5% of the general population and up to 30% of dementia cases (Zaccai et al., 2005; Mueller et al., 2017). PDD is highly prevalent among individuals with Parkinson’s disease (PD), with estimates suggesting that up to 75% of PD patients surviving 10 years and 83% of those who survive 20 years develop PDD (Aarsland et al., 2003; Hely et al., 2008; Aarsland and Kurz, 2010). The prevalence of dementia in PD patients is thought to be 3–6 times higher than the general population (Aarsland et al., 2003; Emre et al., 2007).

These disorders share many clinical, anatomic, and neuropathologic characteristics (Gomperts, 2016; Koga et al., 2021). Patients with each disorder suffer from executive dysfunction, cognitive impairment, hallucinations, and parkinsonism. Both diseases are defined neuropathologically by Lewy bodies (LB), cytoplasmic neuronal inclusions containing α-synuclein (α-syn), in the limbic and cortical regions of the brain. The similarities between the diseases have led some to suggest that they may exist on a common pathophysiologic spectrum.

Modeling the diseases and validating animal models for human Lewy Body Dementia pose a challenge for two main reasons. First, establishing a model of PD or DLB that recapitulates the full spectrum of the disease remains elusive. As will be described in detail below, there is often great heterogeneity in both pathological findings and nervous system regions affected among individual patients with either disorder, while pathogenesis can also be multifactorial. Second, testing cognition in animals with the inability to perform the complex functions that are impaired in humans as well as a lacking of anatomical complexity necessitates rigorous development of animal behavioral paradigms. In this review, we will first describe the current theories for cognitive decline in human Lewy-Body Dementia. We will then explore progress to date in recreating pathology in animals to model cognitive phenotypes seen in humans.

## Part I: understanding cognitive decline in human lewy-body dementia

### Clinical phenotypes

While there is heterogeneity in the cognitive profile of PDD patients, a typical pattern of executive dysfunction, deficits in concentration and attention, memory impairment, and flawed visuo-spatial abilities exists (Emre et al., 2007; Kalbe et al., 2008; Irwin et al., 2013). Specifically, executive function impairment leads to worsened task planning, task initiation, pattern recognition, and concept and rule forming abilities (Litvan et al., 1991; Paolo et al., 1995; Emre et al., 2007; Irwin et al., 2013). Attention and concentration deficits manifest as impaired spontaneous and focused attention, difficulty maintaining concentration, and characteristically fluctuates throughout the day (Ballard et al., 2002; Noe et al., 2004; Kemps et al., 2005; Emre et al., 2007; Kalbe et al., 2008). Poor executive function is thought to contribute to a predominantly recall-related memory impairment in PDD, (Kemps et al., 2005, Kalbe et al., 2008, Godefroy et al., 2010) though memory encoding and storage are affected as well (Higginson et al., 2005; Emre et al., 2007). Considerable evidence reveals that visuo-spatial and perceptive abilities are also impaired in PDD patients (Hovestadt et al., 1987; Bradley et al., 1989; Waterfall and Crowe, 1995; Mosimann et al., 2004; Irwin et al., 2013). The variability in clinical presentation among PDD patients may be reflective of the heterogeneity in neuropathologic patterns in PD (Hurtig et al., 2000; Jellinger and Korczyn, 2018).

Patients with DLB share many clinical characteristics with PDD patients, though there are a few notable differences. The most apparent difference between the two is the timing of cognitive decline, as DLB patients experience cognitive decline at the time of diagnosis or shortly after motor symptom development (usually within 1 year), while PDD patients usually have motor symptoms for a substantial period before cognitive decline onset (McKeith and Burn, 2000; McKeith et al., 2005; Emre et al., 2007). Subtle differences in the cognitive profiles of the patients also exist, as DLB patients have been shown to perform worse than PDD patients in attention tasks (measured by verbal memory and delayed recall) and executive function tasks (measured by the cognitive interference “Stroop” test) (Park et al., 2011). The overall rate of cognitive decline is thought to be faster in DLB than either PDD or Alzheimer’s Disease (AD) (Rongve et al., 2016; Kramberger et al., 2017). Furthermore, hallucinations are more frequent in DLB and can occur any time after disease onset, while PDD patients typically develop hallucinations after levodopa replacement therapy (Jellinger and Korczyn, 2018). Rest tremor is less frequent and balance is less affected in DLB than in PDD, though other motor manifestations, such as bradykinesia and rigidity, are similar between entities (Fritz et al., 2016; Jellinger and Korczyn, 2018).

### Neuropathological phenotypes

#### α synuclein patterns

While neurodegeneration of the nigrostriatal basal ganglia circuitry may be responsible for the motor symptoms of PD, the neuropathologic underpinnings of cognitive decline in DLB and PDD remain less clear, as studies have highlighted the importance of cortical LB and Lewy-neurite pathology, concurrent Alzheimer’s disease-like pathology, and involvement in the brain’s cholinergic system in the development of cognitive decline (Braak et al., 2003; Compta et al., 2011; Irwin et al., 2013). Parkinson’s disease is traditionally characterized by neurodegeneration and accumulation of α-syn^1^ in the substantia nigra, and α-syn pathology can be observed throughout the brain (Braak et al., 2003; Bohnen and Albin, 2011; Irwin et al., 2013). Post-mortem neuropathologic studies have revealed the importance of cortical LBs in development of PDD, (Hurtig et al., 2000, Mattila et al., 2000, Harding and Halliday, 2001, Kövari et al., 2003) with specific involvement of the entorhinal cortex, temporal neocortex, anterior cingulate gyrus, and the pre-frontal cortices (Irwin et al., 2012, 2013). Cortical LB burden has also been shown to predict dementia onset and severity in DLB patients (Ruffmann et al., 2016; Ferman et al., 2018). Studies of α-syn aggregates in animal models have long raised the increasingly accepted concept of a prion-like ability for aggregates to spread throughout the nervous system and even from the periphery, although mechanisms of spread are an active area of research (Luk et al., 2012; Masuda-Suzukake et al., 2013; Kovacs et al., 2017; Henderson et al., 2019). Meticulous pathologic analysis of the brains of PD patients, conducted by Braak and others, have led to theories that PD pathogenesis may follow a caudal-to-rostral pattern of spread in at least some patients (Braak et al., 2003). In these patients, pathology was found to start in the brainstem, spread to limbic, sub-cortical structures, before eventually propagating to the cerebral cortex. In fact, the original description of this abnormality by Lewy noted considerable pathology in the basal forebrain, where cholinergic neurons project to regions responsible for many cognitive functions (Lewy, 1913; Bohnen and Albin, 2011) (Box 1). A challenge for modeling these forms of human disease is thus to recreate where possible this concept of pathology propagation in a manner that is consistent with human observations.

BOX 1 **Definition of terms.**

Dopaminergic Neurons: Neurons that produce dopamine as their primary neurotransmitter, characterized by expression of tyrosine hydroxylase (TH), dopamine transporter (DAT), GIRK2 (a G-Protein coupled receptor), and others. These neurons are found in various regions of the brain, including the substantia nigra and the ventral tegmental area.

Cholinergic Neurons: Neurons that release acetylcholine (ACh) as their primary neurotransmitter, characterized by expression of choline acetyl-transferase (ChAT), acetylcholine esterase (AChE), and others. While they are found throughout the brain, they are typical of basal forebrain structures.

Cortical Neurons: Neurons located in the cerebral cortex, the outer layer of the brain responsible for higher cognitive functions. Cortical neurons are morphologically diverse and classified into various types based on their structure, function, and connectivity within the cortical circuitry.

#### Alzheimer’s disease co-pathology

The presence of concurrent AD-like pathology, extracellular amyloid-β (Aβ) plaques and intracellular neurofibrillary tau-tangles, are predictive of PDD (Kalaitzakis et al., 2008; Compta et al., 2011) and LBD (Jellinger and Attems, 2008; Gomperts, 2016). In fact, it is estimated that between one-quarter and one-half of PDD patients have enough AD pathology to meet post-mortem pathologic criteria for diagnosis (Mattila et al., 1998; Galvin et al., 2006; Sabbagh et al., 2009). Some have demonstrated that time onset to dementia in parkinsonian patients is correlated with cortical deposition of both α-syn and Aβ (Ballard et al., 2006). Both α-syn and AD-pathology are likely involved in development of the Lewy-Body Dementia, possibly in an additive or synergistic manner (Irwin et al., 2013; Ferman et al., 2018).

#### Cholinergic degeneration

In addition to these pathologic hallmarks of neurodegeneration in DLB and PDD, impairment of the brain’s cholinergic systems may play a significant role in development of the diseases. Specifically, cholinergic neurons of the basal forebrain (BF), which have projections to the hippocampus, olfactory bulb, cortical mantle, and amygdala, (Bohnen and Albin, 2011; Pasquini et al., 2021) are implicated in PDD and DLB. Neurodegeneration of BF cholinergic neurons (Arendt et al., 1983; Candy et al., 1983; Nakano and Hirano, 1984; Braak et al., 2003) and their targets in the hippocampus (Perry et al., 1994; Tiraboschi et al., 2002; Hall et al., 2014) have been associated with PDD and DLB. This has been supported by positron-emission tomography (PET) studies revealing decrease in cortical cholinergic activity, (Asahina et al., 1995; Bohnen et al., 2003; Hilker et al., 2005; Shimada et al., 2009; Kanel et al., 2020) and the fact that acetylcholinesterase inhibitors, which prevent acetylcholine degradation, have been shown to improve cognitive outcomes in PDD and DLB (McKeith et al., 2000; Reading et al., 2001; Bergman and Lerner, 2002; Fabbrini et al., 2002; Giladi et al., 2003; Beversdorf et al., 2004; Dujardin et al., 2006; van Laar et al., 2011; Dubois et al., 2012; Mori et al., 2012; Wang et al., 2015). While the exact relationship between cholinergic neurodegeneration and the other neuropathologic findings of Lewy Body Dementia is unknown, there is likely a complex interplay of factors resulting in the ultimate clinical phenotype.

#### PDD-DLB pathologic discrepancies

While there is certainly overlap in the pathology seen in PDD and DLB, subtle differences may explain the disparities in cognitive and overall clinical presentation. There is less nigral neurodegeneration in DLB patients compared to PDD patients, which may explain the motor differences seen between the disorders (Tsuboi and Dickson, 2005). Compared with PDD patients, DLB patients have higher α-syn burden in the hippocampus and amygdala (Kalaitzakis et al., 2009; Jellinger and Korczyn, 2018). Additionally, DLB patients have an increase in cortical and subcortical Aβ when compared to PDD patients, specifically with increased Aβ load in the entorhinal cortex, amygdala, and putamen (Hepp et al., 2016). DLB patients also have higher amounts of cortical atrophy compared to PDD patients, most notably in the temporal, parietal, and occipital lobes (Beyer et al., 2007). Collectively, these may account for the cognitive differences seen between patients with PDD and DLB. These findings also emphasize the challenges faced when attempting to model cognitive dysfunction from synucleiopathies in animal models, since not only must the specific mechanism of generating α-syn pathology be considered but also co-pathologies can be critical to the models and may differ in consequences depending upon both levels of expression and location of expression within specific brain regions.

## Part II: toward animal models of synucleinopathies

### Cognitive testing in rodents

Any attempt to model cognitive decline first requires thorough evaluation and validation of behavioral paradigms. The most commonly used tests for gauging cognitive ability in rodents include the Y-maze, the novel object recognition (NOR) test, the Morris Water Maze (MWM), and fear conditioning (Taylor et al., 2010; Tanila, 2018). In the Y-maze, animals are allowed to freely explore a three-armed maze. An unimpaired animal’s inclination to explore novel areas results in a tendency for alternation between the maze’s arms, while an animal with short-term memory impairment would explore the arms of the maze in an uneven manner. In the NOR test, an animal’s tendency to explore unfamiliar objects is again evaluated. The time spent examining a novel object compared to a familiar one is measured over 2 days. Therefore the NOR test can be used as a measure of both short- and long-term memory. In the MWM, animals are trained to find a hidden platform in a pool of water using visual cues. With extended training, a cognitively unimpaired animal would display a decrease in the latency to find the platform and an increase in the time spent around the platform. The MWM is accepted as a measure of spatial-visual memory.^2^ In fear conditioning, animals are conditioned to associate a neutral stimulus with an aversive stimulus. The development, persistence in a new context and unlearning of the conditioned fear response can all be measured. The facets of cognition that these tests measure and their clinical counterparts are listed in Table 1.

**TABLE 1:** Representative human clinical and animal behavioral tests for various cognitive impairments in Lewy Body Dementia.

Clinical deficit	Example human clinical test	Example animal behavioral test
Executive Function	Stroop Interference Test; (Watson and Leverenz, 2010; Park et al., 2011) Tower of London Test (Kalaitzakis et al., 2009)	Motor sequence learning
Task Planning	Tower of London Test (Weintraub et al., 2005; Watson and Leverenz, 2010)	Goal-directed behavior tests
Attention	Trail Making Test (Watson and Leverenz, 2010); Letter/number sorting task (Kalbe et al., 2008)	–
Concentration	Naming months backward (Pagonabarraga et al., 2010)	–
Memory Retrieval	Word Pair Association Learning Task (Kalbe et al., 2008)	MWM; NOR; Fear conditioning
Memory Encoding	Selective reminding test (Chiaravalloti et al., 2014)	NOR; Fear conditioning
Visuospatial Ability	Clock drawing test (Pagonabarraga et al., 2010)	MWM

MWM, Morris Water Maze; NOR, Novel Object Recognition.

Besides the technical challenge in performing these tests, the tests themselves may not be specific to cognitive ability. For example, the Y-maze, MWM, and NOR can be skewed by visual or olfactory dysfunction. Additionally, the MWM and fear conditioning are heavily influenced by an animal’s sensitivity to aversive stimuli and anxiety, which can be heavily affected by pathology in the limbic system, a region known to be affected in synucleinopathies. Furthermore, these tests rely on movement of animals in a behavioral context due to the inability to communicate with rodents, so any motor deficits could confound interpretation of cognitive dysfunction in these models. Finally, many cognitive tests can only be performed once and so choosing a time where the cognitive impairment will be detectable is critical. The order in which cognitive tests are performed also matters, as stress-inducing tests can confound subsequent performance (Rodriguiz and Wetsel, 2006). As such, it may be difficult to assess the order of symptom development as compared to humans.

### Modeling aspects of lewy body dementia in rodents

When it comes to modeling synucleinopathy in rodents, the two aspects of the disease primarily focused on are the loss of dopaminergic innervation in the midbrain and the progressive accumulation of both α-syn and pSyn. To replicate these fundamental pathologic features, researchers have undertaken a multitude of innovative approaches.

#### Toxin models

To date, the most efficient way of achieving dopaminergic denervation is through the use of the catecholaminergic selective toxins MPTP and 6-hydroxydopamine (6-OHDA). When administered systemically (MPTP) or intracerebrally (6-OHDA), these toxins cause an accumulation of oxidative species resulting in death of dopaminergic neurons. While not involving Lewy pathology specifically, due to their selective targeting of the dopaminergic pathway, these models capture the motor impairments associated with midbrain degeneration well. Toxin-treated mice fail to remain on the rotarod as long as their sham treated littermates and display amphetamine desensitization. When 6-OHDA is injected unilaterally, mice display rotation behaviors as an effect of the asymmetric burden of neurodegeneration. These toxin models are also useful in understanding the contribution of the nigro-striatal dopaminergic pathway to cognition. While both models have been shown to induce deficits in memory and attention, there is some evidence that MPTP is better at impairing short-term memory, and that 6-OHDA primarily affects long-term memory (Fan et al., 2021). One drawback to these models, however, is that the cognitive impairment induced by the toxins is not stable long-term, with animals showing recovery a few months post injection (p.i.) (Bezard et al., 2000; Fan et al., 2021). This could indicate the measured cognitive decline is related to the brain’s inflammatory response rather than the neurodegeneration itself. In a rotenone study, depleting or inactivating microglia (with PLX397 or minocycline, respectively) attenuated the cognitive impairments as measured by the MWM, NOR, and passive avoidance tests. The microglial inhibition however, also attenuated the pSyn pathology and neuronal loss, making it difficult to ascertain whether the neuroinflammation itself caused the impairment (Zhang D. et al., 2021). In contrast, inducing neuroinflammation in wildtype mice by systemic administration of lipopoylysaccharide (LPS), activates microglia and causes hippocampal neuronal loss as well as sickness behavior and impairments in the MWM and passive avoidance tests (Zhao et al., 2019).

#### Viral vector models

Viral vector-mediated overexpression of *SNCA*, the gene coding for α-syn, has been shown to result in a local, progressive accumulation of the protein and severe neurotoxicity around the area of injection (Huntington and Srinivasan, 2021). The use of viral vectors to stably overexpress the protein allows for control of the types of cells affected and the degree of pathology by modulating the injection site, serotype, volume and titre of virus delivered as well as promoter and α-syn form (human vs mouse; full-length vs truncated; wildtype vs mutated) expressed. Even though these models have been reported to display cognitive and motor impairments, (Fan et al., 2021) the focal overexpression, the acute neurotoxicity, and the ensuing inflammatory response are not thought to be representative of the neuropathology of synucleinopathies (Volpicelli-Daley et al., 2016). These models have therefore fallen out of favor in recent years. Nonetheless, if the level of α-syn expression influences susceptibility to pathology or co-pathologies, the viral model still can be attractive if dosing, promoter strength or regulation and cell-type specificity can be adjusted to specific applications.

#### Transgenic models

In contrast, transgenic mouse models overexpressing some form of *SNCA*, often display a time-dependent accumulation of α-syn aggregates which more closely mimic the disease. These models rarely achieve levels of neurodegeneration comparable to the toxin models, but they do reflect a genetic form of human disease, characterized by triplication or mutations of the *SNCA* gene leading to overexpression and eventual pathogenesis. Depending on the promoter and *SNCA* variant used, the spatial and neuropathologic signature of the accumulation can be manipulated (Giasson et al., 2002; Freichel et al., 2007; Zhou et al., 2008). The M83 line, which overexpresses the human familial PD-associated A53T form of *SNCA* under the control of the prion promoter, is one of the most popular transgenic models of synucleinopathy (Giasson et al., 2002). When bred homozygous, these mice develop severe motor phenotypes such as trembling, limb paralysis and inability to stand. Prior to motor symptom onset, which is typically described at 7–8 months of age, these mice display diminished nesting behavior and impaired spatial memory as tested by the Y-maze (Paumier et al., 2013). These mice, however, accumulate α-syn in CNS regions not typically associated with human synucleinopathy, such as the cerebellum and the spinal cord, which may be explained by the use of the prion promoter.

Another well characterized transgenic mouse line, line 61, which overexpresses human full-length wild-type *SNCA* under the control of the Thy-1 promoter, also displays progressive accumulation of α-syn, with the highest fold increase described in the thalamus when compared to wildtype littermates (Chesselet et al., 2012). These mice display reduced motility in the open-field test at 14-months of age, around which time a 40% decrease in striatal TH levels are observed. Prior to motor symptom onset, these mice display mild cognitive impairments as measured by the Y-maze and NOR tests. Similar to many other transgenic mouse models, these mice do not display TH cell loss in the nigra, even at 22-months of age. Similarly, other Thy-1-asyn mice also display cognitive impairments in the NOR (Subramaniam et al., 2018).

Another approach, in an effort to more closely mimic accumulation of α-syn in relevant brain regions, is to overexpress the human form of the protein under the control of the mouse’s endogenous *SNCA* promoter, on either a wildtype (Hansen et al., 2013) or mouse *SNCA* knockout background (Janezic et al., 2013). These mice also display a progressive accumulation of α-syn, very mild neurodegeneration, and progressive motor and cognitive phenotypes starting at around 9-months of age. Besides *SNCA*, other genes have been implicated in PD and LBD in GWAS studies. Among the most commonly reported are *PINK1* and *LRRK2*. *PINK1* KO mice have been developed and though they show impaired synaptic plasticity, (Kitada et al., 2007) cognitive impairment hasn’t been as widely reported (Gispert et al., 2009). *LRRK2*G2019S mice on the other hand, have been reported to show impairments in executive function in the touchscreen operant task tests (Cheng et al., 2022; Hussein et al., 2022). A comprehensive review of transgenic models used and the cognitive impairments reported can be found elsewhere (Fan et al., 2021).

#### Preformed fibril models

**FIGURE 1:**
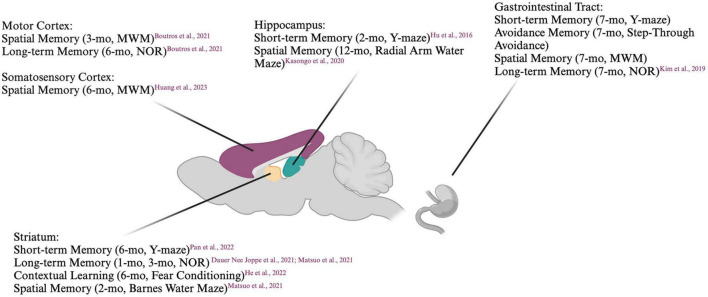
Summary of cognitive deficits elicited based on location of preformed fibril injection. MWM, Morris Water Maze; NOR, Novel Object Recognition. Created with BioRender.com.

A model that has been gaining popularity in recent years is the preformed fibril (PFF) model, in which preformed fibrils of human or mouse α-syn are injected into mice (Chung et al., 2019). Alpha-synuclein can be recombinantly expressed and fibrillization induced, commonly by shaking at 37°C. The resulting α-syn fibrils closely resemble those found in Lewy bodies when examined by electron microscopy (Cremades et al., 2012; Marotta et al., 2021). When added to cells or injected into animals, PFFs have been shown to rapidly induce the templating of endogenous α-syn, resulting in a spatio-temporally defined spread of α-syn aggregates (Polinski et al., 2018). PFFs also cause neurotoxicity, with the severity of the toxicity determined by the α-syn PFF ‘strain’ (Bousset et al., 2013) and cellular subtype, (Alegre-Abarrategui et al., 2019) with nigral dopaminergic neurons showing particular vulnerability. One main advantage of this model is that the targeted pathways and the signature of α-syn pathology can be altered with the injection site. As such, the contributions of different pathways to behavioral deficits can be dissected. Summaries of the cognitive impairments induced with PFF injections are listed in Figure 1 and Table 2.

**TABLE 2:** Summary of conditions and cognitive impairments in rodent preformed fibril models.

Brain Region	Species	Laterality	Coordinates	PFF Strain	Test	Time p.i.	Result	Reference
Striatum	Mouse	Unilateral	AP: +0.2 mm, ML: +2.0 mm, DV :- 2.8mm	5 μg human α-syn	Y-maze	6-months	+	Pan et al., 2022
Striatum	Mouse (prenatal brain iron enrichment study, not significant if only 0 μg iron groups analyzed)	Unilateral	AP: +0.4 mm, ML: -1.8 mm, DV: -3.5 mm	5 μg human α-syn	NOR	3-months	+	Dauer Née Joppe et al., 2021
Striatum	Mouse	Bilateral	AP : +0.2 mm ML : ±2.0 mm DV : -2.6 mm	2 μl/side 300 μM mouse α-syn	Tube test	6-months	+	Stoyka et al., 2020
					Fear conditioning	6-months	+	
Striatum	Mouse	Bilateral	AP : +1.0 mm ML : ±2.0 mm DV : -2.6 mm	2 μl/side 300 μM mouse α-syn	Fear conditioning	6-months	-	Stoyka et al., 2021
Striatum (dorsolateral)	Mouse	Bilateral	AP: +0.98 mm ML: ±2.2 mm DV: -2.6 mm	5 μg/side A53T human α-syn	Satiety-based instrumental training paradigms	3-months	+	He et al., 2022
					Motor sequence learning		+	
Striatum (dorsomedial)			AP: +0.98 mm ML: ±1.2 mm DV: -2.5 mm	5 μg/side A53T human α-syn	Satiety-based instrumental training paradigms	3-months	+	
					Motor sequence learning		-	
Nigra			AP: -3.16 mm ML: ±1.25mm DV: -4.0 mm	5 μg/side A53T human α-syn	Satiety-based instrumental training paradigms	1-month	+	
					Motor sequence learning		+	
Striatum	Mouse	Bilateral	AP: +0.8 mm ML: +2.0 mm DV: +2.6 mm	5 μg/side human ATTO550-α-syn	Y-maze	1-month	-	Matsuo et al., 2021
					NOR	1-month	+	
					Barnes maze	2-months	+	
					Passive avoidance step-through	2-months	+	
Hippocampus	Mouse	Bilateral	AP: -2.2 mm ML: ±1.5 mm DV: -2.3 mm	5 μg/side A53T human α-syn	Y-maze	2-months	+	Hu et al., 2016
Hippocampus	Rat	Bilateral	AP: –3.6 mm ML = ±1.8 mm DV : –3.6 mm	7.5 μg/side mouse α-syn	MWM	3- and 12- months	-	Kasongo et al., 2020
					2-radial arm water maze	12-months	+	
Cortex (motor)	Mouse	Bilateral	AP: –1.0 mm ML: ±1.5 mm DV: -0.3 mm	5 μg/side mouse α-syn	MWM	3-months	+	Boutros et al., 2021
					NOR	6-months	+	
Cortex (medial prefrontal)	Mouse	Bilateral	AP: +1.8 mm ML: ± 0.5 mm DV: -1.8 mm	10 μg/ side human α-syn & AAV-SNCA in medial prefrontal cortex & VTA & basal forebrain	interval timing	6-months	-	Zhang Q. et al., 2021
Cortex (somatosensory) & Striatum	Mouse	Unilateral	AP: +0.26 mm ML : +2.0 mm DV: - 1.5 mm & -3.0 mm	5 μg human α-syn (total)	MWM	6-months	+	Huang et al., 2023
Medial Forebrain Bundle	Rat	Unilateral	AP: +4.0mm, ML: +1.2 mm DV: +7.5 mm (below dura)	30 μg	MWM	4-months	-	Pang et al., 2022
Pyloric Stomach and Duodenum	Mouse	-	-	25 μg mouse α-syn	MWM	7-months	+	Kim et al., 2019
					Y-maze	7-months	+	
					NOR	7-months	+	
					Step-through passive avoidance	7-months	+	

AP, Anterior-posterior; ML, Medial-lateral; DV, Dorsal-ventral; AAV, Adeno-associated virus; SNCA, α-synuclein gene; VTA, Ventral tegmental area;MWM, Morris Water Maze; NOR, Novel Object Recognition; p.i., Post injection; PFF, Preformed fibrils, PFF.

##### Striatum

Mice injected unilaterally in the striatum have been reported to display impaired short-term memory as measured by the Y-maze (Pan et al., 2022) and impaired long-term memory in the NOR test, (Dauer Née Joppe et al., 2021) albeit several months p.i.. When injected bilaterally into the striatum, mouse PFFs have been shown to induce accumulation of pSyn not only in the striato-nigral pathway but also in connected areas such as the prefrontal cortex and amygdala. Six months p.i., these mice have been reported to display diminished social dominance and impairments in fear conditioning in some (Stoyka et al., 2020) but not all studies (Stoyka et al., 2021) despite a loss of tyrosine hydroxylase (TH) immunoreactivity. The coordinates targeted within the striatum may modulate the development of pathology, as mice injected in the dorsolateral but not dorsomedial striatum showed impaired learning even though both groups of mice displayed impaired goal-directed behavior. When the substantia nigra was targeted, impairments in sequence learning were already apparent at 1-month p.i. (He et al., 2022). The NOR test and the Barnes maze test (another spatial memory test) have been shown to detect impairments in mice injected in the dorsal striatum as early as 60 days p.i., at which point the Y-maze did not. This cognitive impairment was attributed to a decrease in septal GABAergic neurons (Matsuo et al., 2021). This is surprising, as a previous study suggested that the septo-hippocampal pathway was not susceptible to pSyn accumulation post PFF injection into the hippocampus (Luk et al., 2012). Interestingly, two other studies have demonstrated a particular resistance of the (cholinergic) medial septal, but not (GABAergic and glutamatergic) lateral septal neurons to α-syn accumulation when PFFs were injected into the hippocampus (Nouraei et al., 2018; Dues et al., 2023).

##### Hippocampus

Mice with PFFs injected bilaterally in the hippocampus displayed pSyn accumulation near the site of injection, in the dentate gyrus. Two months p.i., a reduction in Y-maze alteration was described, which was attributed to aberrant adenosine A2A receptor signaling, Hu et al. (2016) a pathway involved in both glutamatergic and dopaminergic regulation (He et al., 2022). Similarly, rats with bilateral hippocampal PFF injections displayed accumulation of pSyn in the injected and connected areas with concomitant cognitive impairment detected by the two radial arm water maze (a combination of the Y-maze and MWM), but not with the MWM (Espa et al., 2019; Kasongo et al., 2020).

##### Cortex

Cortical structures have also been targeted with PFFs. Bilateral injection of PFFs into the motor cortex induced impairment of spatial memory as measured by MWM at 3-months p.i. After 6-months, these same mice displayed impairment in memory as measured by NOR (Boutros et al., 2021). While no habituation was observed in the open field test, this may be explained by hyperactivity, which has previously been reported in PD-mouse models (Freichel et al., 2007). When PFFs were injected bilaterally into the medial prefrontal cortex 6-months after AAV-induced overexpression of human *SNCA* in the medial prefrontal cortex, ventral tegmental area, and basal forebrain, pSyn accumulation was induced not only in the PFF-injected areas, but also in the striatum, substantia nigra, and entorhinal cortex. Six months after the PFF injections, however, the mice displayed no differences in interval timing compared to controls. Although it seemed the PFF injected mice displayed a slight decrease in mobility and discrimination in the open field and NOR tests, respectively, these differences did not reach statistical significance (Zhang Q. et al., 2021). Six months after human PFFs were unilaterally injected into the right somatosensory cortex and dorsal striatum, mice displayed cognitive impairments as measured by the MWM (Huang et al., 2023). In contrast, pSyn in the hippocampus and frontal cortex induced by a unilateral injection of PFFs in the medial forebrain bundle in rats did not result in a measurable cognitive impairment in the MWM up to 120 days p.i. (Pang et al., 2022).

##### Peripheral injections

In an attempt to model a “body first” synucleinopathy, mouse PFFs injected in the gut have been shown to produce a progressive, caudal-rostral accumulation of pSyn in the brainstem, cortex, hippocampus and striatum (Kim et al., 2019). This was accompanied by cognitive impairments as measured by the MWM, Y-maze, NOR, and step-through passive-avoidance test 7-months p.i.

#### Alpha-synuclein co-pathologies

Considering the prevalence of co-pathology in patients, another strategy for PFF-induction of synucleinopathy is the use of α-syn PFFs in Aβ, tau, or APOE mouse models. Injecting A53T PFFs unilaterally into the right frontal cortex has been shown to improve the spatial memory deficits observed in APP/PS1 mice with Aβ accumulation. Surprisingly, in that same study, wildtype mice injected with the PFFs also showed a statistically significant improvement in the MWM compared to PBS-injected controls (Hao et al., 2018). In contrast, in a study using a different model of Aβ pathology, 5xFAD mice injected unilaterally in the hippocampus with mouse α-syn PFFs displayed cognitive impairment, as measured by the Y-maze, starting at 3-months p.i. and continued to worsen over time. These mice showed a progressive increase in pSyn accumulation compared to wildtype mice injected with the PFFs. While the pSyn was primarily contained to the hippocampus, hypothalamus, and cortex at 9-months p.i. in wildtype mice, the pSyn load was already ubiquitous throughout the brain at 4.5-months p.i. in the 5xFAD mice. The PFF injected 5xFAD mice also displayed an increase Aβ and hyperphosphorylated tau burden compared to PBS injected 5xFAD mice (Bassil et al., 2020). Even though tau has been shown to co-localize with α-syn at presynaptic terminals, there have been conflicting reports on the neuroprotective effects of knocking out tau in the PFF model (Stoyka et al., 2021; Pan et al., 2022) with one study suggesting a unidirectional relationship with α-syn as a modulator of tau pathology (Bassil et al., 2020). In the A53T transgenic model, knocking out tau not only reduced neuronal loss but attenuated the memory deficits associated with the model (Singh et al., 2019). Both phosphorylated tau and pSyn have been shown to be upregulated in the MPTP model of neurodegeneration (Hu et al., 2020). Finally, two separate studies have demonstrated a modulatory effect of *APOE* genotype on α-syn pathology. While *APOE2* showed a neuroprotective effect compared to *APOE3* and even *APOE* KO, *APOE4* exacerbated α-syn pathology burden, both in the A53T mouse (Davis et al., 2020) and in the AAV-induced overexpression model (Zhao et al., 2020). The α-syn *APOE4* mice also demonstrated a faster cognitive decline compared to the other *APOE* genotypes.

#### Confounding factors and potential explanations of discrepancies

The discrepancies in results between studies may be attributed to one of several factors. The strain of PFFs used, which is dictated by the aggregating conditions and specific α-syn variant (mouse vs human, wildtype vs familial-associated point-mutations (A53T, A30P, etc.), may influence outcome. Although many groups use the same behavioral tests, the protocols for these tests (for instance, whether they are performed during the light or dark cycle) and therefore also the readouts vary between groups. The number and sex of animals used also affects whether any potential impairment reaches statistical significance, which in turn affects how differences in behavior are reported. Additionally, the timepoint at which the cognitive behaviors are evaluated can influence what is observed. If a late timepoint is used, many areas of the brain may be burdened with pSyn and as such conclusions about the studied pathway can be confounded. If an early timepoint is used, however, the pSyn pathology may be restricted to the area of injection and closely connected pathways, but the acute inflammation accompanying the injections may confound any observed cognitive impairment. While cholinergic pathology is thought to underlie cognitive impairments in humans, many mouse models have failed to recapitulate cholinergic degeneration in mice. This could be due to the key differences in cholinergic neurons in terms of distribution, functionality and structural roles in rodents as compared to primates (Disney and Robert, 2019). Acute systemic inflammation is known to cause reversible cognitive impairments (Skelly et al., 2019) but inflammation has also been suggested as a key driver in α-syn pathology (Kim et al., 2022).

## Conclusion

In this review, we have explored the clinical manifestations and underlying pathologic mechanisms of Lewy Body Dementia, encompassing both Parkinson’s Disease Dementia and Dementia with Lewy Bodies. Additionally, we have assessed the different strategies by which researchers have attempted to emulate the cognitive deficits of these diseases in animal models. The cognitive decline in Lewy Body Dementia emerges from a complex interplay of α-syn propagation, concomitant AD-like Aβ and tau pathology, and loss of neurotransmitter tone, which may work in an additive or synergistic manner. While these pathologic hallmarks are diffuse by the time cognitive decline becomes evident, careful investigations highlight pivotal regions that drive the progression to dementia, including the pre-frontal cortex, temporal neo-cortex, hippocampus, and cholinergic neurons of the basal forebrain.

While the discrepancies in reported pathology in the different animal models pose challenges to researchers, it is important to remember that the symptomatology of PDD and LBD patients can also vary widely. An enhanced understanding of the pathophysiology of these diseases as well as the now large repertoire of animal models available to researchers should aide in developing breakthrough treatments if used correctly. Improved testing will also be key in elucidating relevant cognitive impairments and potential therapies for them. Tests for higher executive cognitive function, such as attentional set shifting tasks and odor span tasks, have not been routinely performed but would be interesting in light of the specific executive dysfuntion seen in patients.

As of now, there is no ultimate animal model—the choice to be made depends on the question to be answered with each model having a unique profile. The tools to study these challenging disorders continue to evolve, and the emergence of multiple hit models offer the promise to better replicate PDD and LBD pathology in a near-physiologic manner.

## Author contributions

GW: Conceptualization, Data curation, Formal analysis, Methodology, Project administration, Writing – original draft, Writing – review and editing. CH: Conceptualization, Data curation, Formal analysis, Methodology, Project administration, Writing – original draft, Writing – review and editing. MK: Conceptualization, Funding acquisition, Project administration, Resources, Supervision, Validation, Writing – original draft, Writing – review and editing.

## Footnotes

1 Recent mass spectroscopy-based studies have shown that modified α-syn with phosphorylation of serine residue 129 (pSyn) is the dominant version of the protein within LBs and may in fact be responsible for aggregation and pathogenesis in PDD and LBD.

2 The Barnes maze test is a similar test of spatial memory and navigation, though involves a circular platform with several holes, one of which leads to escape from an elevated platform.
